# Anti-survival of motor neuron antibodies in rheumatic and musculoskeletal diseases: prevalence, clinical associations, and biomarker potential, with novel insights into disease activity in SLE

**DOI:** 10.1186/s41232-025-00399-w

**Published:** 2025-12-02

**Authors:** Yuki Imai, Masaru Takeshita, Koji Suzuki, Hiroyuki Fukui, Kazunori Furuhashi, Kotaro Matsumoto, Jun Kikuchi, Keiko Yoshimoto, Yuko Kaneko

**Affiliations:** Division of Rheumatology, Department of Internal Medicine, Keio University School of Medicine, 35 Shinanomachi, Shinjuku-Ku, Tokyo, Japan

**Keywords:** Autoantibodies, Biomarkers, Lupus erythematosus, systemic, Mixed connective tissue disease, SMN complex proteins

## Abstract

**Background:**

Anti-survival of motor neuron (SMN) antibodies have recently been identified in rheumatic and musculoskeletal diseases (RMDs), notably mixed connective tissue disease (MCTD). However, their immunological characteristics, prevalence, and clinical relevance beyond MCTD remain poorly understood. This study aimed to elucidate the clinical significance of anti-SMN antibodies in a wide spectrum of RMDs.

**Methods:**

We assessed anti-SMN antibodies and antibody-producing cells using Western blotting and immunofluorescence staining with recombinant SMN complexes. Serum anti-SMN antibody titers were measured using a recombinant SMN complex-bound magnetic bead assay in 906 serum samples from patients with 16 types of RMDs and healthy controls. Clinical associations and treatment responses were analyzed.

**Results:**

Western blotting using patients’ sera confirmed SMN-specific antibodies. Immunofluorescence staining identified anti-SMN antibody-producing plasma cells in an MCTD patient’s lymph node. Anti-SMN antibodies were detected in 36.7% of MCTD, 10.6% of systemic lupus erythematosus (SLE), and 2.4% of systemic sclerosis patients, while none of the healthy controls were positive. Antibody titers were higher against the SMN complex than individual components, highlighting the importance of the complex structure. In MCTD, antibody positivity was strongly correlated with interstitial lung disease (90.9% vs. 36.8%, *P* = .013). In SLE, antibody-positive patients had significantly lower white blood cell counts and complement levels, higher anti-ds-DNA antibody titer, and higher prevalence of serositis (35.0% vs. 11.3%), gastrointestinal involvement (15.0% vs. 2.4%), nephritis (70.0% vs. 30.4%), and higher median SLE Disease Activity Index scores (19.5 vs. 5.0) compared to antibody-negative patients (all *P* < .05). Antibody titers decreased after treatment (− 71.5% at 3 months, *P* = .010) and increased upon relapse.

**Conclusions:**

Anti-SMN antibodies are prevalent in MCTD and SLE. Consistent with prior studies, their titers in MCTD were associated with distinct clinical features. Importantly, we newly demonstrate that in SLE, anti-SMN antibody levels correlate with immune complex–related manifestations and disease activity, providing a novel and clinically significant insight. These findings highlight their potential as biomarkers for disease stratification, organ involvement, and monitoring disease progression in RMDs.

**Supplementary Information:**

The online version contains supplementary material available at 10.1186/s41232-025-00399-w.

## Introduction

Autoantibodies are critical in diagnosing rheumatic and musculoskeletal diseases (RMDs), predicting organ damage, and assessing disease activity. For instance, anti-U1-ribonucleoprotein (RNP) antibodies are essential for the classification of mixed connective tissue disease (MCTD), whereas anti-Sm and anti-ds-DNA antibodies support diagnosis and indicate organ involvement and disease activity in systemic lupus erythematosus (SLE) [1–3].

Anti-survival of motor neuron (SMN) antibody was initially identified in idiopathic inflammatory myopathies (IIM) in 2011 [4]. These antibodies target the SMN complex, which is essential for the assembly of small nuclear ribonucleoproteins (snRNPs) involved in pre-mRNA splicing [5, 6]. This complex consists of 9 proteins (SMN1, Gemin2 to Gemin8, and unr-interacting protein [UNRIP]) and is ubiquitously expressed and localized in the cytoplasm and nucleus, particularly in Cajal bodies [7]. Anti-SMN antibodies are frequently found in conjunction with anti-U1-RNP antibodies in patients with MCTD, SLE, IIM, and systemic sclerosis (SSc) [4, 8–11]. Notably, 36% to 59% of patients with MCTD are reported to be positive for anti-SMN antibodies, which have been linked to clinical manifestations including interstitial lung disease (ILD), pulmonary arterial hypertension (PAH), myocarditis, and lower gastrointestinal involvement [8, 9]. These associations hint at the possible involvement of anti-SMN antibodies in disease pathogenesis, addressing the lack of activity markers in MCTD and anti-ds-DNA antibody-negative SLE.

However, the prevalence, clinical significance, and immunological characteristics of anti-SMN antibodies in diverse RMDs have not been thoroughly explored. In this study, we aimed to investigate their frequency across multiple RMDs and assess associated clinical features. We additionally examined immunological aspects, including antigen reactivity profiles, autoantibody overlap, and the cellular origin of antibody production. Ultimately, we sought to clarify the potential utility of anti-SMN antibodies as biomarkers in autoimmune diseases.

## Methods

### Human samples

Serum samples were obtained from 81 healthy controls (HCs) and 825 patients with RMDs at clinically determined time points between April 2012 and February 2024 at Keio University Hospital. Samples were preferably collected at disease onset or relapse. Patients were classified based on established diagnostic or classification criteria for MCTD (*n* = 30), SLE (*n* = 188), SSc (*n* = 126), IIM (*n* = 72), primary Sjögren’s disease (*n* = 136), rheumatoid arthritis (*n* = 91), spondyloarthritis (*n* = 9), anti-neutrophil cytoplasmic antibody-associated vasculitis (*n* = 76), polyarteritis nodosa (*n* = 4), giant cell arteritis (*n* = 40), polymyalgia rheumatica (*n* = 52), Takayasu arteritis (*n* = 28), relapsing polychondritis (*n* = 14), IgG4-related disease (*n* = 5), Behçet’s disease (*n* = 5), and Still’s disease (*n* = 5). Supplementary Table S1 shows the criteria used in the cohort. Among them, 667 patients (80.8%) had isolated RMDs.

Clinical parameters including demographic features, laboratory results, clinical manifestations, and treatments were retrieved from medical records. ILD was diagnosed in all patients by chest computed tomography (CT), confirmed independently by both radiologists and rheumatologists. PAH was diagnosed by cardiologists when all of the following hemodynamic criteria were met: (1) mean pulmonary arterial pressure (mPAP) > 25 mmHg, (2) pulmonary arterial wedge pressure (PAWP) ≤ 15 mmHg, and (3) pulmonary vascular resistance (PVR) > 3 Wood units. Disease severity and activity were evaluated using the severity classification from the Japanese clinical practice guidelines for MCTD 2021 [12] and Systemic Lupus Erythematosus Disease Activity Index 2000 (SLEDAI-2K) for SLE [13]. Relapse in both MCTD and SLE was defined as an increase of more than 3 points in Safety of Estrogens in Lupus Erythematosus National Assessment-SLEDAI (SELENA-SLEDAI) [14], or elevation in British Isles Lupus Assessment Group (BILAG) score [15] to A or B, accompanied by treatment intensification.

### Recombinant proteins

The SMN complex and its individual components (SMN1, Gemin2–8, UNRIP), U1-RNP (SNRNP70, SNRPA, SNRPC), and Sm protein (SNRPD1) were cloned into pcDNA3.4 vectors (Thermo Fisher Scientific, USA) with streptavidin-binding peptide (SBP) tags and either green fluorescent protein (GFP) or polyhistidine (His) tags at the N-terminus of the antigens. Anti-U1-RNP antibodies typically bind to SNRPB/B’ antigens, leading to cross-reactivity to Sm protein (SNRPB/B’, SNRPD–G) [16]. To minimize false-positive results, SNRPD1 was utilized specifically for anti-Sm antibody detection. In the SMN complex, only the SMN1 protein carried SBP and His tags. These vectors were transfected into human embryonic kidney (HEK) 293 T cells (Riken BRC, Japan) using polyethyleneimine (Polysciences, USA). After 2 days of culture, the cells were lysed in Tris-buffered saline containing 1% Triton X-100 (TBSTx) and a protease inhibitor cocktail (Fujifilm Wako, Japan). The cell lysates were cleared by centrifugation at 16,000 × *g* at 4 ℃ for 15 min, and the supernatant was aliquoted and stored at − 80 ℃ until use.

### Western blotting and silver staining

The supernatant of cell lysates containing SBP-tagged SMN complexes or individual SMN antigens was incubated with Dynabeads M-280 Streptavidin (Thermo Fisher Scientific) for 30 min. After washing, the bound proteins were eluted by boiling in 1 × sodium dodecyl sulfate (SDS) sample buffer (Bio-Rad, USA) at 95 ℃ for 5 min. The purified proteins were separated by sodium dodecyl sulfate-polyacrylamide gel electrophoresis (SDS-PAGE) using a 12.5% gel, transferred onto polyvinylidene difluoride (PVDF) membranes, and blotted by Strep-Tactin horseradish peroxidase (HRP)-conjugated protein (Bio-Rad), or visualized by silver staining (APRO Science, Japan).

### Immunofluorescence staining

Immunofluorescence staining using a fluorescence-conjugated autoantigen was performed as previously described [17] with the following modifications. Lymph node tissue was collected from the left axilla of a patient with MCTD who underwent a biopsy for diagnostic purpose. OCT-embedded frozen sections were incubated with 5 μg/mL of an anti-CD138 antibody (unconjugated, mouse IgG1; BioLegend) and 20 μg/mL of SBP- and His-tagged SMN complexes purified using Streptavidin Sepharose High Performance (Cytiva Japan, Japan) at 4 ℃ overnight. Sections incubated without SMN complexes served as negative controls. After washing, the sections were stained with 4 μg/mL of anti-mouse IgG1 antibody (Alexa Fluor 594, goat; Thermo Fisher Scientific) and 2 μg/mL of anti-His-tag antibody (Alexa Fluor 488, mouse IgG2; MBL, Japan) at room temperature for 1 h. After additional washing, the sections were mounted using Vectashield Mounting Medium with DAPI (Vector Laboratories, USA). Antibody-producing cells were visualized using an LSM 980 confocal microscope (Zeiss, Germany) and analyzed using ImageJ2 (version 2.14.0).

### Antigen-binding bead assay

The titers of autoantibodies were measured using antigen-binding bead assay as previously described [18] with the following modifications. To prevent cross-reactivity with other antigens, the supernatant of cell lysates containing U1-RNP and Sm antigens was treated with 20 μg/mL Ribonuclease A (Nippon Gene, Japan) at 37 ℃ for 30 min. The supernatant containing SMN, U1-RNP, and Sm antigens was then incubated with Dynabeads M-280 Streptavidin for 30 min. After washing, the antigen-bound beads were incubated with subject sera (1:300 dilution) for 20 min, washed again, and then stained with an anti-human IgG-Fc antibody (Alexa Fluor 647, goat F(ab’)_2_ fragment; Jackson ImmunoResearch, USA). Antibody titers were quantified as median fluorescence intensity (MFI) of Alexa Fluor 647 using a FACSVerse flow cytometer (BD Biosciences, USA) and analyzed with FlowJo software (version 10.9.0; BD Biosciences). A sample was considered positive if the MFI exceeded 10 times (anti-SMN: 1,050 MFI; anti-U1-RNP: 3,940 MFI) or 5 times (anti-Sm: 610 MFI) the outlier threshold of HCs, calculated as the 75th percentile + 1.5 × interquartile range (IQR). To ensure high specificity, higher cutoff values were adopted, as lower thresholds (mean + 2 SD) would have yielded unrealistically high positivity rates due to low variance in healthy controls and cross-reactivity with related antibodies.

### Western blotting with patient sera

The SMN proteins were separated by SDS-PAGE, and transferred onto PVDF membranes. Membranes were blocked with PVDF Blocking Reagent for Can Get Signal (Toyobo, Japan) for 1 h, and incubated with patient sera (1:300 dilution) from patients with MCTD for 1 h. Autoantibodies binding to SMN antigens were detected using HRP-conjugated anti-human IgG-Fc (goat F(ab’)_2_ fragment; Jackson ImmunoResearch) for 1 h. After washing, signals were visualized using ImmunoStar LD (Fujifilm Wako).

### Immunoprecipitation followed by Western blotting

Dynabeads Protein G (Thermo Fisher Scientific) were incubated with sera (1:100 dilution) from two SMN-high-titer patients (> 30,000 MFI), two SMN-low-titer patients (1,000–2,500 MFI), one SMN-negative patient and one healthy control (100–500 MFI) at 4 ℃ for 2 h. SBP- and His-tagged SMN complexes overexpressed in 293 T cell extracts were added and incubated overnight at 4 ℃. After washing, bound proteins were eluted by boiling at 95 ℃ for 5 min, separated by SDS-PAGE, and transferred to PVDF membranes. Membranes were probed with Strep-Tactin HRP-conjugate, and signals were visualized using ImmunoStar LD (Fujifilm Wako).

### Statistical analysis

Continuous variables were compared using Student’s t-test or Mann–Whitney U test, depending on data normality. Categorical variables were compared using the chi-square test or Fisher’s exact test, depending on sample size. Correlations between 2 variables were analyzed using Spearman’s rank correlation coefficient. Antibody titers before and after treatment were compared using the Wilcoxon signed-rank test. A *P*-value < 0.05 was considered statistically significant. All statistical analyses were performed using R version 4.4.1.

### Ethics approval

This study was approved by the Ethics Committee of Keio University School of Medicine (approval number: 20130246) and conducted in accordance with the Declaration of Helsinki. Written informed consent was obtained from all participants.

## Results

### Purification of recombinant SMN complex

**Fig. 1:**
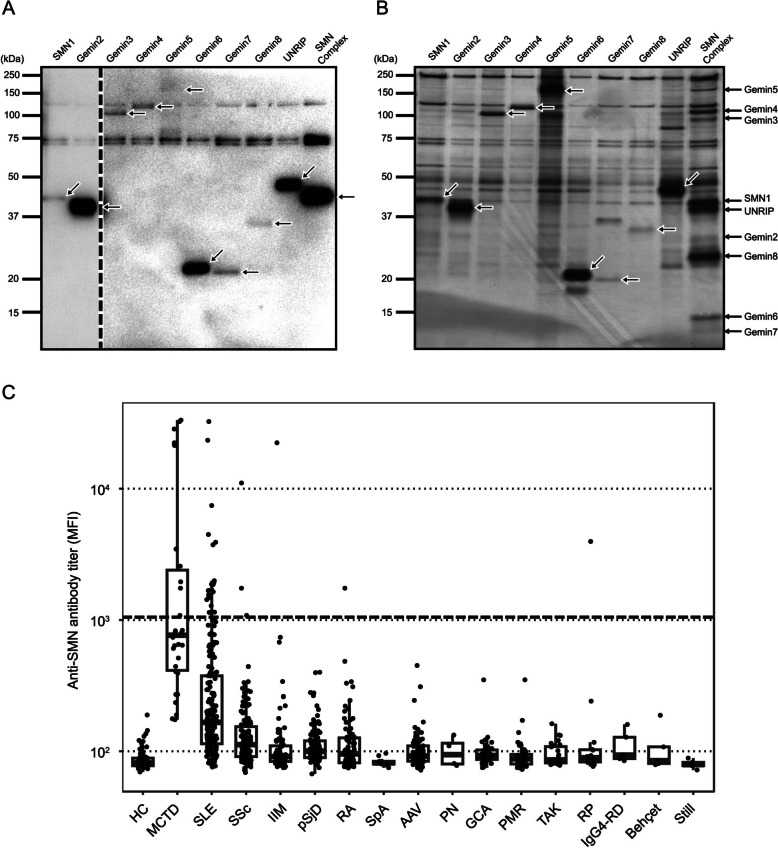
Confirmation of SMN antigens and anti-SMN antibody titers in various RMDs. **A**, **B** Purified SBP-tagged individual SMN antigens or SMN complexes were resolved by SDS-PAGE and **A** blotted by Strep-Tactin HRP conjugate or **B** silver stained. Arrows indicate target proteins. The dashed line indicates lanes that are derived from the same membrane but that have different contrasts. **C** Anti-SMN antibody titers (log scale) were measured using SMN complex-bound beads and flow cytometry in serum samples from 906 individuals. The cohort included patients with RMDs: mixed connective tissue disease (MCTD; *n* = 30), systemic lupus erythematosus (SLE; *n* = 188), systemic sclerosis (SSc; *n* = 126), idiopathic inflammatory myopathy (IIM; *n* = 72), and others (*n* = 409), as well as healthy controls (HC; *n* = 81). The thick dashed line represents the positivity cutoff value (1,050 MFI)

As has been shown in previous studies by our group and others, autoantibodies recognize the conformational structure of autoantigens well [18–20]. Therefore, we first tried to purify the SMN proteins as a complex form. The expression vector of SBP-tagged SMN1 protein was co-transfected with those of other SMN constituent proteins without a protein tag. From the co-transfected cell lysate, proteins were purified using streptavidin beads and visualized by Western blotting and silver staining, along with single protein expressing cell lysates (Fig. 1A, B). We confirmed that all of the SMN constituent proteins were purified via SBP-tagged SMN1, indicating that these proteins form a complex.

### Serum anti-SMN antibody titers across various RMDs

Next, we measured the serum anti-SMN antibody titers in patients with various RMDs by antigen-binding bead assay using purified recombinant SMN complex. The characteristics of individuals including 81 HCs and 825 patients with RMDs are shown in Table 1. The comprehensive autoantibody profiles of the RMDs, together with the coexistence rates of anti-SMN antibodies and other autoantibodies, are provided in Supplementary Table S2. The log-scale antibody titers across various RMDs are shown in Fig. 1C. The highest titers were in patients with MCTD followed by patients with SLE, and the positivity rates were 36.7% and 10.6%, respectively. Anti-SMN antibodies were rarely observed in other RMDs, with only 3 cases in SSc and 1 case each in idiopathic inflammatory myopathy, rheumatoid arthritis, and relapsing polychondritis.

**Table 1:** Clinical parameter and anti-SMN antibody titers

	**Healthy controls** **(*n* = 81)**		**All RMDs**^**a**^ **(*n* = 825)**		**Mixed connective tissue disease** **(*n* = 30)**		**Systemic lupus erythematosus** **(*n* = 188)**		**Systemic sclerosis**^**b**^ **(*n* = 126)**		**Idiopathic inflammatory myopathy**^**c**^ **(*n* = 72)**	
Age (years), mean (SD)	44.0	(14.3)	58.3	(16.9)	48.2	(16.4)	46.9	(16.0)	63.4	(12.6)	60.7	(13.9)
Female, *n* (%)	63	(77.8)	678	(82.2)	26	(86.7)	161	(85.6)	115	(91.3)	59	(81.9)
Anti-SMN titer (MFI), median (IQR)	81.6	(77.6–88.7)	102.0	(86.2–145.0)	766.0	(413.5–2,415.5)	166.0	(114.0–375.3)	112.5	(91.0–154.0)	91.2	(83.7–109.0)
Anti-SMN titer > 1,050 (MFI), *n* (%)	0	(0.0)	32	(3.9)	11	(36.7)	20	(10.6)	3	(2.4)	1	(1.4)
Anti-U1-RNP titer > 3.940 (MFI), *n* (%)	0	(0.0)		N/A	27	(90.0)	40	(21.3)	8	(6.3)	3	(4.2)
Anti-Sm titer > 610 (MFI), *n* (%)	0	(0.0)		N/A	5	(16.7)	40	(21.3)	2	(1.6)	2	(2.8)
Immunomodulatory therapy naïve, *n* (%)	81	(100.0)	458	(55.5)	11	(36.7)	67	(35.6)	99	(78.6)	30	(41.7)
Patients with isolated RMD^a^, *n* (%)	0	(0.0)	667	(80.8)	17	(56.7)	150	(79.8)	91	(72.2)	52	(72.2)
	**Primary Sjögren’s disease** **(*n* = 136)**		**Rheumatoid arthritis** **(*n* = 91)**		**Spondyloarthritis**^**d**^ **(*n* = 9)**		**ANCA-associated vasculitis**^**e**^ **(*n* = 76)**		**Polyarteritis nodosa** **(*n* = 4)**		**Giant cell arteritis** **(*n* = 40)**	
Age (years), mean (SD)	60.4	(15.5)	61.9	(15.3)	56.2	(17.1)	64.4	(13.1)	59.8	(10.5)	73.8	(6.3)
Female, *n* (%)	129	(94.9)	80	(87.9)	5	(55.6)	52	(68.4)	4	(100.0)	25	(62.5)
Anti-SMN titer (MFI), median (IQR)	100.0	(89.4–120.0)	95.6	(81.6–126.5)	81.6	(80.0–83.9)	91.8	(84.0–110.0)	95.4	(80.0–115.8)	92.9	(86.6–102.3)
Anti-SMN titer > 1,050 (MFI), *n* (%)	0	(0.0)	1	(1.1)	0	(0.0)	0	(0.0)	0	(0.0)	0	(0.0)
Immunomodulatory therapy naïve, *n* (%)	125	(91.9)	23	(25.3)	3	(33.3)	48	(63.2)	4	(100.0)	29	(72.5)
Patients with isolated RMD^a^, *n* (%)	136	(100.0)	65	(71.4)	9	(100.0)	65	(85.5)	3	(75.0)	24	(60.0)
	**Polymyalgia rheumatica** **(*n* = 52)**		**Takayasu arteritis** **(*n* = 28)**		**Relapsing polychondritis** **(*n* = 14)**		**IgG4-related disease** **(*n* = 5)**		**Behçet’s disease** **(*n* = 5)**		**Still’s disease** **(*n*** **= 5)**	
Age (years), mean (SD)	76.4	(7.2)	42.0	(12.9)	59.6	(13.8)	59.6	(10.4)	33.6	(7.5)	56.4	(21.9)
Female, *n* (%)	38	(73.1)	17	(60.7)	6	(42.9)	3	(60.0)	2	(40.0)	4	(80.0)
Anti-SMN titer (MFI), median (IQR)	87.0	(79.8–93.1)	86.0	(81.5–108.5)	89.2	(82.9–102.8)	87.9	(86.0–93.5)	84.8	(80.7–108.0)	79.1	(77.6–82.3)
Anti-SMN titer > 1,050 (MFI), *n* (%)	0	(0.0)	0	(0.0)	1	(7.1)	0	(0.0)	0	(0.0)	0	(0.0)
Immunomodulatory therapy naïve, *n* (%)	27	(51.9)	10	(35.7)	3	(21.4)	3	(60.0)	2	(40.0)	2	(40.0)
Patients with isolated RMD^a^, *n* (%)	35	(67.3)	28	(100.0)	14	(100.0)	2	(40.0)	5	(100.0)	5	(100.0)

^a^RMDs included diseases listed above as well as secondary Sjögren’s disease (*n* = 81)

^b^Systemic sclerosis included diffuse cutaneous (*n* = 24) and limited cutaneous (*n* = 102) subsets

^c^Idiopathic inflammatory myopathy included anti-synthetase syndrome (*n* = 20), immune mediated necrotizing myopathy (*n* = 5), polymyositis (*n* = 13) and dermatomyositis (*n* = 34)

^d^Spondyloarthritis included axial spondyloarthritis (*n* = 4), psoriatic arthritis (*n* = 2), and SAPHO (Synovitis, Acne, Pustulosis, Hyperostosis, Osteitis) syndrome (*n* = 3)

^e^ANCA-associated vasculitis included eosinophilic granulomatosis with polyangiitis (*n* = 25), granulomatosis with polyangiitis (*n* = 26), and microscopic polyangiitis (*n* = 25). *ANCA*, anti-neutrophil cytoplasmic antibody; *IQR*, interquartile range; *N/A*, not applicable; *MFI*, median fluorescence intensity; *SD*, standard deviation; *SMN*, survival of motor neuron

### Reactivities of anti-SMN antibodies to the SMN constituent antigens

**Fig. 2:**
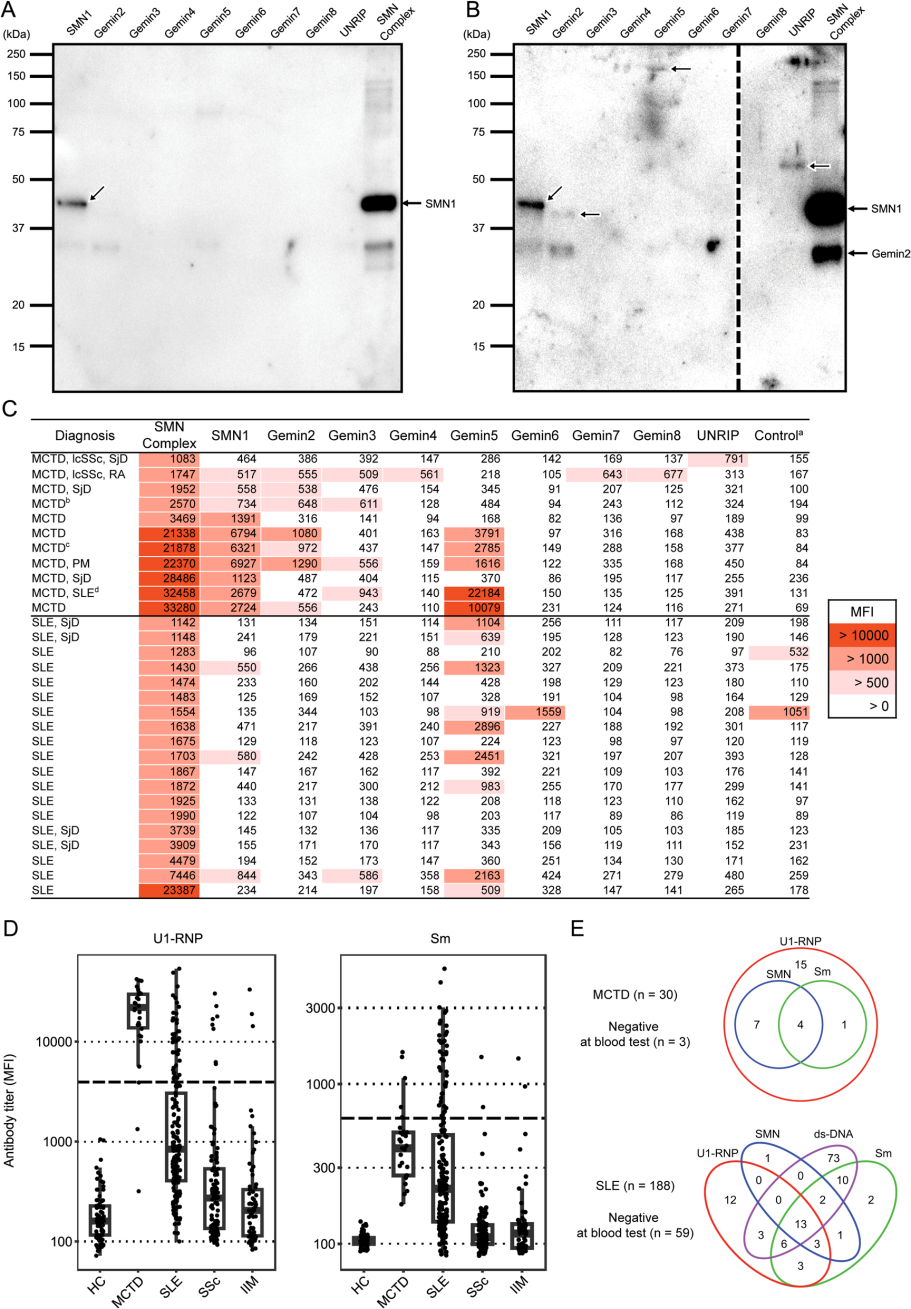
Target region of anti-SMN antibodies and their coexistence with other antibodies. **A**, **B** Purified SBP-tagged individual SMN antigens or SMN complexes were resolved by SDS-PAGE and blotted by patients’ sera. **A** Serum antibodies from a 44-year-old female patient recognized SMN1 antigen, whereas **B** serum antibodies from a 65-year-old female patient recognized multiple antigens (arrows). The dashed line indicates lanes that are derived from the same membrane but that have different contrasts. **C** The reactivity of serum anti-SMN antibodies against the recombinant SMN complex and its individual constituent proteins were assessed by flow cytometry using single antigen-binding beads. The analysis included anti-SMN antibody-positive MCTD and SLE patients. Numbers within the heatmap represent the antibody titers (MFI). **D** Anti-U1-RNP and anti-Sm antibody titers (log scale) were measured in serum samples by flow cytometry using recombinant U1-RNP- and Sm-binding beads. Thick dashed lines indicate the cutoff values for positivity. **E** Numbers represent patients categorized according to positivity for autoantibodies. ^a^Control consisted of SBP-GFP proteins. ^b^The patient lymph node sample was used for immunohistochemistry in Fig. 3D. ^c, d^The patient serum samples were used for Western blotting in **A** and **B**, respectively

Subsequently, we investigated the target region of the anti-SMN antibodies using sera positive for anti-SMN antibodies in 2 different experiments. First, to examine reactivity against linear epitopes, we performed Western blotting by applying patient sera to the PVDF membrane shown in Fig. 1A, onto which individual SMN constituent proteins had been transferred. As shown in Fig. 2A, B, anti-SMN antibodies in the sera of 2 cases recognized the linear epitope of SMN1, and, in 1 case, additionally Gemin2, Gemin5, and UNRIP. Second, to examine the conformational epitopes, we performed an antigen-binding bead assay using recombinant antigens that had not been denatured or dried. The heatmap shows serum antibody titers against each constituent protein among the MCTD and SLE patients who were positive for anti-SMN complex antibody (Fig. 2C). The titers against the complex form of SMN were higher than those against its constituent antigens, suggesting the importance of conformational structure for antibody recognition. Additionally, the titers of anti-SMN1 and anti-Gemin2 were higher in MCTD than in SLE, whereas the titers of anti-Gemin5 were elevated in both MCTD and SLE. Some patients showed weak reactivity to SBP-GFP proteins serving as control antigens, but not to all SBP- and His-tagged antigens, suggesting the potential presence of anti-GFP antibodies. Immunoprecipitation followed by Western blotting further provided additional support for the bead assay findings, confirming reactivity to SMN antigens in SMN-high- and SMN-low-titer patients, but not in SMN-negative patient or healthy control (Supplementary Fig. S1). Full-length original gel and blot images are presented in Supplementary Fig. S2.

### Association of anti-SMN antibodies and other autoantibodies

To investigate the association between the presence of anti-SMN antibodies and that of other known autoantibodies, reactivity to U1-RNP and Sm antigens was measured using the antigen-binding bead assay in patients with MCTD, SLE, SSc, IIM, and HC (Fig. 2D). For anti-U1-RNP antibody, 90.0% of MCTD and 21.3% of SLE patients were positive with a cutoff value of 3,940 MFI. For anti-Sm antibody, 16.7% of MCTD and 21.3% of SLE patients were positive with a cutoff value of 610 MFI. None of the HC tested positive for these autoantibodies. Some patients tested negative, possibly due to decreased titers following treatment modification. Venn diagrams for autoantibody presence in patients with MCTD and SLE revealed that all of the anti-SMN antibody-positive MCTD patients were also positive for anti-U1-RNP antibody, whereas 95% of anti-SMN antibody-positive SLE patients were also positive for anti-Sm antibody (Fig. 2E). Among patients with anti-ds-DNA negative SLE, where specific disease activity markers are absent, 4 patients tested positive for anti-SMN antibodies.

**Fig. 3:**
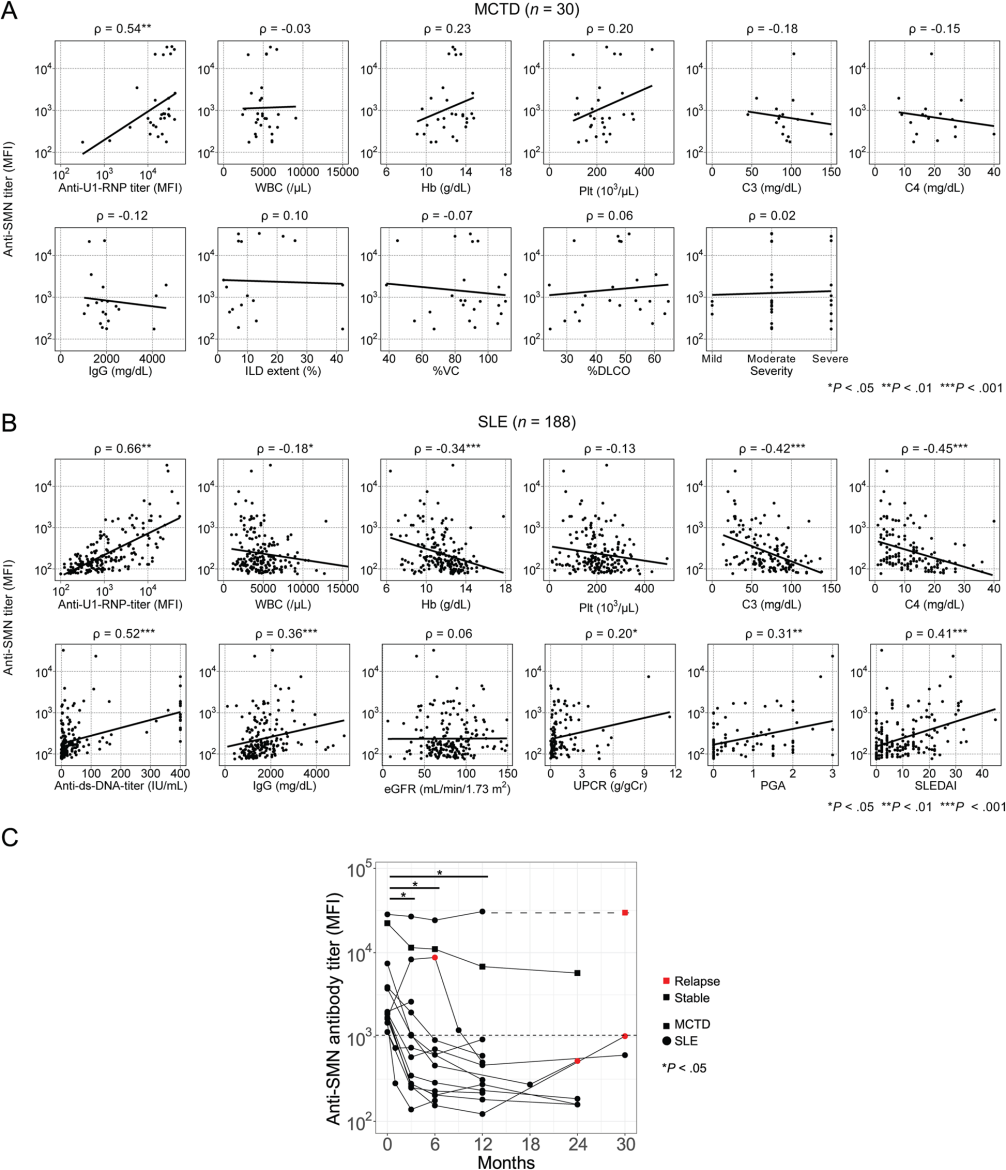
Clinical significance of anti-SMN antibodies. **A**, **B** Scatter plots showing correlations between anti-SMN antibody titers (log scale, measured by flow cytometry) and key clinical metrics in patients with MCTD (**A**) and SLE (**B**). Spearman correlation coefficients (*ρ*) and corresponding *P*-values are indicated for each comparison. Solid lines indicate lines of best fit. **C** Transition of anti-SMN titers following treatment intensification. The analysis included anti-SMN antibody-positive patients: 2 patients with MCTD (square dots) and 13 patients with SLE (circle dots). In a case where relapse occurred at a time point without titer measurement, a dashed line was used to connect data points for visual clarity. Red dots represent relapses observed during the 3-year follow-up period. Changes in antibody titers from baseline to post-treatment were evaluated using the Wilcoxon signed-rank test

### Clinical significance of anti-SMN antibody in MCTD and SLE

We further analyzed the clinical differences between anti-SMN antibody-positive (n = 11) and antibody-negative (*n* = 19) patients with MCTD (Table 2). Age, newly onset versus relapse, sex, blood counts, IgG and complement levels, and treatment status were comparable between the groups. Regarding active clinical symptoms, manifestations of SLE (81.8% vs. 42.1%) and SSc (100.0% vs. 73.7%) were more frequently observed in anti-SMN antibody-positive patients than in antibody-negative patients. Additionally, the coexistence of all 3 disease manifestations, including SLE, SSc, and polymyositis/dermatomyositis, tended to be more common in anti-SMN-positive patients (27.3% vs. 5.3%). Notably, 90.9% of anti-SMN positive patients had ILD, whereas only 36.8% of antibody-negative patients had ILD (*P* = 0.013). Scatter plots exhibited no associations between anti-SMN antibody titer and major clinical values, ILD extent, %vital capacity (%VC) in patients with ILD, or severity (Fig. 3A). When cumulative organ involvement was considered, ILD was the only manifestation that remained significantly more frequent in the anti-SMN-antibody-positive group (Supplementary Table S3).

**Table 2:** Clinical characteristics of MCTD patients stratified by anti-SMN antibody titer

	Negative, *n* = 19 (SMN ≤ 1,050 MFI)		Positive, *n* = 11 (SMN > 1,050 MFI)			*P*-value
Age at blood test (years), mean (SD)	46.4	(18.1)		51.2	(14.5)	.434
Disease duration (months), median (IQR)	42.5	(2.2–215.1)		158.1	(4.4–196.9)	.846
Newly onset, *n* (%)	4	(21.1)		3	(27.3)	>.999
Female, *n* (%)	16	(84.2)		10	(90.9)	>.999
Body mass index (kg/m^2^), median (IQR)	21.4	(19.5–24.4)		19.8	(18.5–22.0)	.268
White blood cell counts (/μL), median (IQR)	4,900.0	(4,200.0–6,100.0)		4,800.0	(3,775.0–5,400.0)	.462
Lymphocyte (/μL), median (IQR)	1,053.2	(759.0–1,255.2)		1,290.3	(1,071.4–1,610.9)	.164
Hemoglobin (g/dL), median (IQR)	12.7	(11.1–13.9)		12.5	(11.8–13.0)	.696
Platelet (10^3^/μL), median (IQR)	223.0	(173.0–246.5)		226.0	(186.5–292.8)	.582
eGFR (mL/min/1.73 m^2^), median (IQR)	73.0	(62.5–98.5)		88.5	(76.5–96.8)	.291
C-reactive protein (mg/L), median (IQR)	1.3	(0.6–3.7)		0.9	(0.3–2.0)	.323
Immunoglobulin G (mg/dL), median (IQR)	1,905.5	(1,750.5–2,071.3)		1,881.0	(1,461.3–3,605.0)	.741
Complement 3 (mg/dL), median (IQR)	90.0	(85.0–98.0)		91.5	(74.8–102.3)	.692
Complement 4 (mg/dL), median (IQR)	18.0	(14.0–26.0)		18.0	(14.8–21.5)	.865
Anti-SMN antibody titer (MFI), median (IQR)	513.0	(271.0–695.0)		21,338.0	(2,261.0–25,428.0)	<.001
Positivity for anti-recombinant U1-RNP antibody, *n* (%)	16	(84.2)		11	(100.0)	.279
Positivity for anti-recombinant Sm antibody, *n* (%)	1	(5.3)		4	(36.4)	.047
Positivity for anti-ds-DNA antibody, *n* (%)	2	(10.5)		3	(27.3)	.204
Positivity for anti-SS-A antibody, *n* (%)	10	(52.6)		6	(54.5)	.740
Raynaud’s phenomenon, *n* (%)	18	(94.7)		10	(90.9)	>.999
Puffy fingers or swollen hands, *n* (%)	9	(47.4)		5	(45.5)	>.999
Pulmonary arterial hypertension, *n* (%)	5	(26.3)		2	(18.2)	>.999
Aseptic meningitis, *n* (%)	0	(0.0)		0	(0.0)	N/A
Trigeminal neuropathy, *n* (%)	0	(0.0)		1	(9.1)	.367
SLE active manifestations, *n* (%)	8	(42.1)		9	(81.8)	.057
Polyarthritis, *n* (%)	3	(15.8)		5	(45.5)	.104
Lymphadenopathy, *n* (%)	2	(10.5)		2	(18.2)	.611
Malar rash, *n* (%)	1	(5.3)		3	(27.3)	.126
Pericarditis or pleuritis,* n* (%)	4	(21.1)		1	(9.1)	.626
Leukopenia (≤ 4,000/μL) or thrombocytopenia (≤ 100,000/μL), *n* (%)	3	(15.8)		3	(27.3)	.336
SSc active manifestations, *n* (%)	14	(73.7)		11	(100.0)	.129
Sclerodactyly, *n* (%)	11	(57.9)		7	(63.6)	>.999
Interstitial lung disease, *n* (%)	7	(36.8)		10	(90.9)	.013
Interstitial lung disease extent (%), median (IQR)	8.0	(6.0–12.5)		9.0	(7.0–20.0)	.845
Esophageal dysmotility or dilatation, *n* (%)	5	(26.3)		2	(18.2)	.952
PM/DM active manifestations, *n* (%)	4	(21.1)		3	(27.3)	>.999
Myositis, *n* (%)	4	(21.1)		1	(9.1)	.626
Elevated levels of myogenic enzymes, *n* (%)	2	(10.5)		3	(27.3)	.419
All manifestations of SLE, SSc and PM/DM, *n* (%)	1	(5.3)		3	(27.3)	.125
Disease severity (mild, moderate, severe), *n* (%)	3 (15.8), 10 (52.6), 6 (31.6)			0 (0), 7 (63.6), 4 (36.4)		.380
Immunomodulatory therapy naïve, *n* (%)	7	(36.8)		4	(36.4)	>.999
Prednisolone usage at blood test, *n* (%)	10	(52.6)		4	(36.4)	.466
Prednisolone dosage at blood test (mg/day), median (IQR)	2.0	(0.0–5.5)		0.0	(0.0–4.0)	.497
Immunosuppressant usage at blood test, *n* (%)	4	(21.1)		4	(36.4)	.417
Concurrent RMDs, *n* (%)	7	(36.8)		6	(54.5)	.575

*eGFR* estimated glomerular filtration rate, *IQR* interquartile range, *N/A* not applicable, *MCT*D mixed connective tissue disease, *MFI* median fluorescence intensity, *PM/DM* polymyositis/dermatomyositis, *RMD* rheumatic and musculoskeletal disease, *RNP* ribonucleoprotein, *SD* standard deviation, *SLE* systemic lupus erythematosus, *SMN* survival of motor neuron, *SSc* systemic sclerosis

We also analyzed the clinical differences between anti-SMN antibody-positive (*n* = 20) and antibody-negative (*n* = 168) patients with SLE (Table 3). Anti-SMN-positive patients exhibited significantly lower white blood cell (WBC) counts (3,500 vs. 4,650/μL), lower hemoglobin (9.8 vs. 11.8 g/dL), lower complement (C)3 and C4 (34.0 vs. 72.5 mg/dL and 6.0 vs. 12.0 mg/dL, respectively), and higher anti-ds-DNA antibody titer (92.2 vs. 11.7 IU/mL) compared with anti-SMN-negative patients. Additionally, they had an increased prevalence of fever (35.0% vs. 13.7%), serositis (35.0% vs. 11.3%), gastrointestinal involvement (15.0% vs. 2.4%), and nephritis (70.0% vs. 30.4%), and higher median SLEDAI (19.5 vs. 5.0) (all *P* < 0.05). A similar trend was observed when the analysis was limited to newly diagnosed patients with SLE (Supplementary Table S4). Twelve newly onset anti-SMN-positive patients exhibited significantly lower WBC counts, lower hemoglobin, lower C3 and C4, and higher anti-ds-DNA antibody titer compared with 49 anti-SMN-negative patients (all *P* < 0.05). They also tended to show a more frequent prevalence of fever, serositis, gastrointestinal involvement, nephritis, and higher median SLEDAI scores, although some differences did not reach statistical significance due to small sample size. A scatter plot revealed that anti-SMN antibody titer was significantly and negatively correlated with WBC counts (*ρ* = − 0.18), hemoglobin (*ρ* = − 0.34), C3 (*ρ* = − 0.42), and C4 (*ρ* = − 0.45), and positively correlated with anti-ds-DNA antibody titer (*ρ* = 0.52), IgG (*ρ* = 0.36), patient global assessment (*ρ* = 0.31), and SLEDAI (*ρ* = 0.41) (Fig. 3B, all *P* < 0.05). These results suggest that anti-SMN antibodies are associated with specific disease phenotypes and/or disease activities.

**Table 3:** Clinical characteristics of SLE patients stratified by anti-SMN antibody titer

	**Negative, *n* = 168** **(SMN ≤ 1,050 MFI)**		**Positive, *n* = 20** **(SMN > 1,050 MFI)**		***P*-value**
Age at blood test (years), mean (SD)	47.4	(15.9)	42.1	(17.1)	.199
Disease duration (months), median (IQR)	71.0	(3.0–196.5)	7.5	(0.0–116.5)	.104
Newly onset, *n* (%)	49	(29.2)	12	(60.0)	.011
Female, *n* (%)	144	(85.7)	17	(85.0)	>.999
Body mass index (kg/m^2^), median (IQR)	20.4	(18.6–23.0)	19.5	(17.9–20.8)	.084
White blood cell counts (/μL), median (IQR)	4,650.0	(3,375.0–6,025.0)	3,500.0	(2,600.0–4,700.0)	.017
Lymphocyte (/μL), median (IQR)	852.0	(535.0–1,245.0)	723.7	(506.0–829.5)	.061
Hemoglobin (g/dL), median (IQR)	11.8	(10.7–12.8)	9.8	(9.1–11.6)	.001
Platelet (10^3^/μL), median (IQR)	202.0	(159.0–262.0)	175.0	(121.0–229.0)	.088
eGFR (mL/min/1.73 m^2^), median (IQR)	79.5	(64.0–96.0)	75.5	(59.2–89.8)	.370
C-reactive protein (mg/L), median (IQR)	0.7	(0.2–4.6)	0.6	(0.3–1.6)	.918
Immunoglobulin G (mg/dL), median (IQR)	1,522.5	(1,158.0–1,978.0)	1,877.0	(1,408.5–2,384.0)	.050
Complement 3 (mg/dL), median (IQR)	72.5	(53.0–90.0)	34.0	(29.0–58.0)	<.001
Complement 4 (mg/dL), median (IQR)	12.0	(6.0–19.0)	6.0	(3.5–10.5)	.006
Immune complex-C1q (μg/ml), median (IQR)	0.0	(0.0–3.2)	7.2	(0.0–8.3)	.006
Anti-SMN antibody titer (MFI), median (IQR)	152.5	(108.0–244.0)	1,785.0	(1,480.8–3,781.5)	<.001
Positivity for anti-recombinant U1-RNP antibody, *n* (%)	24	(14.3)	16	(80.0)	<.001
Positivity for anti-recombinant Sm antibody, *n* (%)	21	(12.5)	19	(95.0)	<.001
Positivity for anti-ds-DNA antibody, *n* (%)	92	(54.8)	15	(75.0)	.137
Anti-ds-DNA antibody titer (IU/mL), median (IQR)	11.7	(3.5–39.7)	92.2	(19.4–400)	<.001
Positivity for anti-SS-A antibody, *n* (%)	104	(61.9)	14	(70.0)	.549
Positivity for anti-cardiolipin antibody, *n* (%)	56	(33.3)	10	(50.0)	.280
Active manifestations					
Fever, *n* (%)	23	(13.7)	7	(35.0)	.033
Mucocutaneous, *n* (%)	45	(26.8)	10	(50.0)	.058
Neuropsychiatric, *n* (%)	15	(8.9)	1	(5.0)	>.999
Musculoskeletal, *n* (%)	37	(22.0)	7	(35.0)	.309
Cardiorespiratory, *n* (%)	9	(5.4)	1	(5.0)	>.999
Serositis, *n* (%)	19	(11.3)	7	(35.0)	.011
Gastrointestinal, *n* (%)	4	(2.4)	3	(15.0)	.028
Nephritis, n (%)	51	(30.4)	14	(70.0)	.001
Classification of renal pathology (III/IV, III/IV + V, V), *n* (%)	21 (41.2), 11 (21.6), 8 (15.7)		9 (64.3), 3 (21.4), 1 (7.1)		.488
Hemolytic anemia, *n* (%)	7	(4.2)	4	(20.0)	.019
SLEDAI, median (IQR)	5.0	(2.0–14.0)	19.5	(6.0–28.2)	<.001
SLICC/ACR damage index at baseline, median (IQR)	0.0	(0.0–1.0)	0.0	(0.0–1.0)	.493
Immunomodulatory therapy naïve, *n* (%)	55	(32.7)	12	(60.0)	.025
Prednisolone usage at blood test, *n* (%)	101	(60.1)	8	(40.0)	.097
Prednisolone dosage at blood test (mg/day), median (IQR)	2.5	(0.0–6.2)	0.0	(0.0–6.8)	.322
Immunosuppressant usage at blood test, *n* (%)	79	(47.0)	4	(20.0)	.030
Concurrent RMDs, *n* (%)	33	(19.6)	5	(25.0)	.788
Concurrent Sjögren’s disease, *n* (%)	25	(14.9)	4	(20.0)	.520
Concurrent antiphospholipid syndrome, *n* (%)	10	(6.0)	0	(0.0)	.603

*eGFR* estimated glomerular filtration rate, *IQR* interquartile range, *MFI* median fluorescence intensity, *RMD* rheumatic and musculoskeletal disease, *RNP* ribonucleoprotein, *SD* standard deviation, *SLE* systemic lupus erythematosus, *SLEDAI* SLE Disease Activity Index, *SLICC/ACR* Systemic Lupus International Collaborating Clinics/American College of Rheumatology, *SMN* survival of motor neuron

To examine the transition of anti-SMN antibody titer after treatment intensification, we measured antibody titers every 3 to 6 months in 2 MCTD and 13 SLE cases. Treatment intensification was defined as prednisolone titration from 0.5 to 1 mg/kg with or without immunosuppressants, and performed at month 0 for all patients except one patient who relapsed at month 6. As shown in Fig. 3C, anti-SMN antibody titers declined by 71.5% (IQR, 41.6–83.3%; *P* = 0.010) from baseline after 3 months of treatment and became negative after 6 months in most patients. Within 3 years after the first sample collection, 4 of 15 patients experienced relapse. Notably, all exhibited increases in anti-SMN antibody titer at the time of relapse compared with the previous visit.

### Detection of anti-SMN antibody-producing cells in the lymph node

**Fig. 4:**
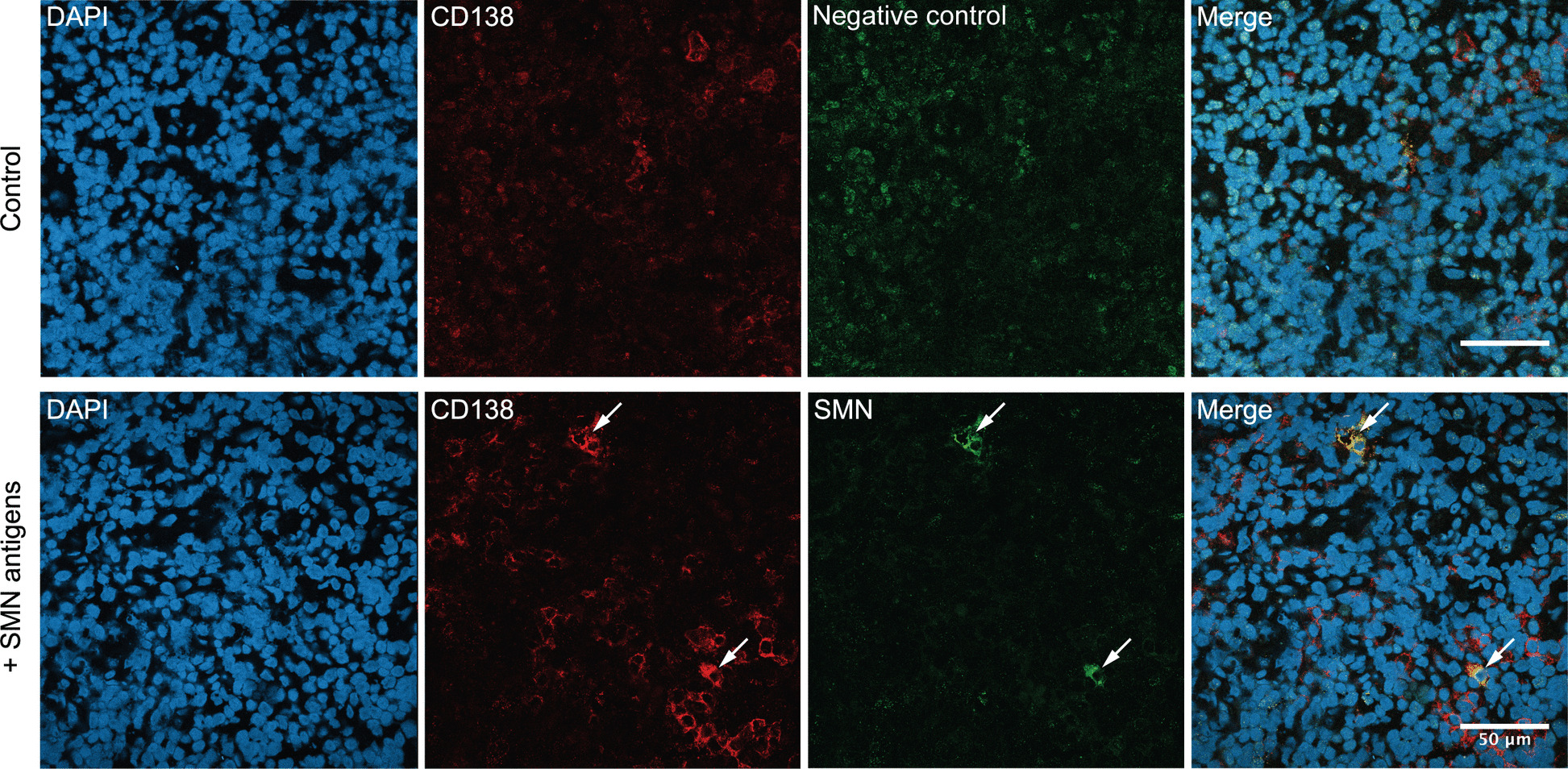
Identification of anti-SMN complex antibody-producing cells in a lymph node. Representative immunofluorescence staining of frozen lymph node tissue obtained from a 23-year-old male patient with MCTD. Negative control is shown as immunofluorescence without SMN antigens (top), while immunofluorescence with SMN antigens is shown (bottom). Sections were stained with DAPI (blue), anti-CD138 antibody (red), and recombinant SMN complexes (green). Anti-SMN complex antibody-producing plasma cells were localized primarily in extrafollicular regions (arrows)

Finally, we analyzed the local production of anti-SMN antibodies in patient tissue. Immunofluorescence staining was performed on a lymph node biopsy from a patient with MCTD. The biopsy had been obtained for diagnostic purposes and revealed reactive lymphoid hyperplasia. As shown in Fig. 4, we observed cells that were positive for both the SMN complex and anti-CD138 antibody predominantly in extrafollicular areas, indicating that these cells were plasma cells producing anti-SMN antibodies. In contrast, the negative control showed only weak nonspecific staining without any cytoplasmic signal indicative of true positivity.

## Discussion

We identified anti-SMN antibody-producing plasma cells within lymph nodes, evaluated their prevalence and titers across 906 specimens, and revealed distinct reactivity profiles against individual SMN components. Consistent with previous reports, anti-SMN antibodies were strongly correlated with ILD in MCTD and showed novel associations with disease severity markers in SLE, including responsiveness to treatment.

This large cohort study revealed that anti-SMN antibodies were found in > 1 patient only among those with MCTD (11 patients, 36.7%), SLE (20 patients, 10.6%), and SSc (3 patients, 2.4%). The observed prevalence of anti-SMN antibodies in MCTD (36%–59%) and in SLE (8%) aligns with previously reported data [8, 9]. In contrast, anti-SMN antibodies were not detected in other isolated RMDs, including rheumatoid arthritis, vasculitis, and autoinflammatory conditions. This finding is consistent with the absence of prior reports in these diseases and suggests high specificity for MCTD and SLE. Anti-SMN antibody titers in anti-SMN-negative MCTD patients were higher than those in HC. Because the SMN complex binds Sm proteins [6, 21], sera containing anti-U1-RNP or anti-Sm antibodies may show weak reactivity to SMN complex-coated magnetic beads via endogenous Sm/RNP molecules bound to SMN proteins. To ensure specificity, we set the cutoff value for anti-SMN antibody positivity at 10 times the outlier threshold determined from HC. Before sample collection, 44.5% of the patients had already received immunomodulatory therapy, which could potentially influence the positivity rate of anti-SMN antibodies, as antibody titer decreases after treatment. However, anti-SMN antibodies were not detected in most diseases, even among the treatment-naïve patients. Therefore, it is unlikely that the absence of anti-SMN antibody-positive cases in other diseases was substantially affected by treatment status.

Previous reports indicated that anti-SMN antibodies could be detected by immunoprecipitation, but not by Western blotting, in certain sera [4, 9]. Our study demonstrated higher antibody titers to the complex form of the SMN than to its constituent antigens, implying the importance of the structural integrity for antigen recognition. These observations indicate that the use of the native SMN complex may enhance detection signal in antibody assays, although their clinical application remains technically challenging. Using individual antigen-binding beads, patients with MCTD showed higher antibody titers against SMN1 and Gemin2 than did patients with SLE. Given earlier reports linking Gemin4 with ILD [22], further research into correlations between individual SMN antigens and specific clinical phenotypes or organ involvement is warranted.

We identified plasma cells as the source of anti-SMN antibodies. Although this observation is derived from a single case and its generalizability is limited, it provides valuable direct evidence supporting the in vivo relevance of anti-SMN antibody production. The precise mechanism underlying SMN-specific antibody production remains unclear, as is the case for most autoantibodies. The production of autoantibodies is thought to be influenced by various genetic and environmental factors, including HLA polymorphisms [23], viral infections [4], post-translational modifications of autoantigens [24], and impaired clearance of apoptotic materials [25]. Because anti-SMN antibodies commonly coexist with anti-U1-RNP or anti-Sm antibodies, and considering the spatial proximity of the SMN complex to the snRNP complex, which is recognized by anti-U1-RNP and anti-Sm antibodies, epitope spreading from these initially recognized antigens is a plausible hypothesis. Longitudinal measurement of serum antibodies from the preclinical phase to the post-onset period is needed to elucidate the temporal sequence of anti-RNP, anti-Sm, and anti-SMN antibody appearance.

Anti-SMN antibody-positive patients with RMDs exhibit a higher prevalence of ILD, PAH, myositis, and SSc-related symptoms compared with anti-SMN antibody-negative patients [8, 9, 26]. The present study also found numerically higher prevalence rates of ILD, myositis, and SSc manifestations in anti-SMN antibody-positive MCTD patients. However, only ILD reached statistical significance due to the limited sample size and treatment modification. On another front, this study is the first to report the clinical relevance of anti-SMN antibody titers in SLE, demonstrating correlations with key clinical metrics, including cytopenia, anti-ds-DNA antibody titer, complement reduction, various organ involvement, and SLEDAI. The observed decline in C3 and C4 and the increase in anti-ds-DNA antibody titer and complex-C1q levels in anti-SMN antibody-positive SLE patients suggest immune complex formation and increased complement consumption. Additionally, these patients showed a higher prevalence of serositis, gastrointestinal involvement, and nephritis, potentially related to immune complexes [27–30]. Conversely, neuropsychiatric symptoms and antiphospholipid syndrome (APS), which have limited immune complex involvement [31, 32], were infrequent. The previous study comparing patients with SLE reported that those with low levels of both C3 and C4, indicating activation of the classical complement pathway, had higher rates of arthritis, serositis, nephritis, and higher SLEDAI scores [28]. In contrast, patients with low C3 alone, reflecting activation of the alternative pathway, more often had APS. Anti-SMN antibody levels might thus reflect immune complex–mediated pathologies, especially through the classical pathway in SLE.

Most patients showed decreased anti-SMN antibody titers following treatment intensification, whereas 4 patients who experienced relapse exhibited increased titers compared to previous visits. These findings may help address the unmet need for disease-specific activity markers in MCTD and anti-ds-DNA-negative SLE, where reliable biomarkers are currently lacking. In prior reports, anti-SMN antibodies have been suggested to aid in the diagnosis of MCTD, and correlate with organ involvement and prognosis [8, 9]. Our results indicate that anti-SMN antibodies may serve as a useful biomarker for disease stratification, assessment of disease activity and organ involvement, and prediction of relapse and prognosis in patients with SLE, in addition to those with MCTD.

Our study has several limitations. First, the number of patients was limited for certain diseases such as polyarteritis nodosa, IgG4-related disease, and autoinflammatory disorders. Although anti-SMN antibodies have not been detected in these conditions, their presence cannot be entirely ruled out in future surveillance studies. Second, a population of patients had received treatment before sample collection, which may have affected the anti-SMN antibody positivity rates. Third, there is currently no standardized assay for measuring anti-SMN antibody titers, and our bead-based assay is challenging to implement in the clinical setting. Nonetheless, the fundamental findings in our study are broadly applicable and could be extended when more feasible assays such as ELISA are developed for routine clinical application. Fourth, longitudinal data on antibody titers after treatment intensification were available in only a subset of patients. Finally, the cohort consisted exclusively of Japanese individuals, which may affect the generalizability of our findings. Nevertheless, a major strength of the present study lies in the measurement of antibody titers against the entire native SMN complex in a large, multi-disease RMD cohort that met validated classification criteria.

## Conclusions

Anti-SMN antibodies exhibit distinct immunological profiles and disease associations, with clear specificity for MCTD and SLE. They are linked to key clinical metrics, including ILD in MCTD and disease activity and organ involvement in SLE, along with treatment responsiveness. Our findings suggest the potential utility of anti-SMN antibodies in stratifying disease phenotypes and predicting clinical outcomes, particularly given their correlation with disease activity in SLE. Further studies are warranted to validate their clinical significance, elucidate underlying immunological mechanisms, and support the development of clinically applicable assays.

## Supplementary Information

Supplementary Material 1.

Supplementary Material 2.

## Data Availability

All data relevant to the study are included in the article.

## Abbreviations

AAV: Anti-neutrophil cytoplasmic antibody-associated vasculitis
ANCA: Anti-neutrophil cytoplasmic antibody
APS: Antiphospholipid syndrome
Behçet: Behçet’s disease
BILAG: British Isles Lupus Assessment Group
CRP: C-reactive protein
CT: Computed tomography
DAPI: 4′,6-Diamidino-2-phenylindole
dcSSc: Diffuse cutaneous systemic sclerosis
DLCO: Diffusing capacity of the lungs for carbon monoxide
DM: Dermatomyositis
dsDNA: Double-stranded DNA
eGFR: Estimated glomerular filtration rate
ELISA: Enzyme-linked immunosorbent assay
FACS: Fluorescence-Activated Cell Sorting
GCA: Giant cell arteritis
GFP: Green Fluorescent Protein
HC: Healthy control
Hb: Hemoglobin
HEK: Human embryonic kidney
HRP: Horseradish peroxidase
IgG4-RD: IgG4-related disease
IIM: Idiopathic inflammatory myopathy
ILD: Interstitial lung disease
IQR: Interquartile range
lcSSc: Limited cutaneous systemic sclerosis
MCTD: Mixed connective tissue disease
MFI: Median fluorescence intensity
mPAP: Mean pulmonary arterial pressure
N/A: Not applicable
PAH: Pulmonary arterial hypertension
PAWP: Pulmonary arterial wedge pressure
PGA: Patient global assessment
PVR: Pulmonary vascular resistance
Plt: Platelet
PM: Polymyositis
PMR: Polymyalgia rheumatica
PN: Polyarteritis nodosa
pSjD: Primary Sjögren’s disease
PVDF: Polyvinylidene difluoride
RA: Rheumatoid arthritis
RMD: Rheumatic and musculoskeletal disease
RNP: Ribonucleoprotein
RP: Relapsing polychondritis
SBP: Streptavidin-binding peptide
SD: Standard deviation
SDS-PAGE: Sodium dodecyl sulfate-polyacrylamide gel electrophoresis
SELENA-SLEDAI: Safety of Estrogens in Lupus Erythematosus National Assessment-SLEDAI
SLE: Systemic lupus erythematosus
SLEDAI: Systemic Lupus Erythematosus Disease Activity Index
SLICC/ACR: Systemic Lupus International Collaborating Clinics/American College of Rheumatology
SMN: Survival of motor neuron
snRNPs: Small nuclear ribonucleoproteins
SpA: Spondyloarthritis
SSc: Systemic sclerosis
Still: Still’s disease
TAK: Takayasu arteritis
TBSTx: Tris-buffered saline containing 1% Triton X-100
UNRIP: Unr-interacting protein
UPCR: Urine protein creatinine ratio
VC: Vital capacity
WBC: White blood cell
