# Supplementary material for: Anti-survival of motor neuron antibodies in rheumatic and musculoskeletal diseases: prevalence, clinical associations, and biomarker potential, with novel insights into disease activity in SLE
Source: Inflamm Regen. 2025 Dec 2;46:1. doi: 10.1186/s41232-025-00399-w (PMC12777490; doi:10.1186/s41232-025-00399-w)
Supplement: Supplementary file 1 — Supplementary Material 1. [file 41232_2025_399_MOESM1_ESM.pdf]

Supplementary Table S1. Diagnostic and classification criteria used for patient inclusion

Diagnosis	Diagnostic and classification criteria
Mixed connective tissue disease	2019 Diagnostic Criteria for Mixed Connective Tissue Disease [S1]
Systemic lupus erythematosus	2012 SLICC Classification Criteria for Systemic Lupus Erythematosus [S2]
	2019 EULAR/ACR Classification Criteria for Systemic Lupus Erythematosus [S3]
Systemic sclerosis	2010 Japanese Ministry of Health, Labor and Welfare Diagnostic Criteria for Systemic Sclerosis [S4]
	2013 ACR/EULAR Classification Criteria for Systemic Sclerosis [S5]
Idiopathic inflammatory myopathies	Bohan and Peter Criteria (1975) [S6]
	Sontheimer Diagnostic criteria for dermatomyositis (1991) [S7]
	2014 Japanese Ministry of Health, Labor and Welfare Diagnostic Criteria for Polymyositis/Dermatomyositis [S8]
	2017 EULAR/ACR Classification Criteria for Adult and Juvenile Idiopathic Inflammatory Myopathies [S9]
Sjögren's disease	Revised Japanese criteria for Sjögren’s syndrome (1999) [S10]
	2016 ACR/EULAR Classification Criteria for Primary Sjögren's Syndrome [S11]
Rheumatoid arthritis	The ACR 1987 Revised Criteria for the Classification of Rheumatoid Arthritis [S12]
	2010 Rheumatoid Arthritis Classification Criteria [S13]
Spondyloarthritis (Axial spondyloarthritis, Psoriatic arthritis, SAPHO syndrome)	Modified New York Criteria for Ankylosing Spondylitis (1984) [S14]
	The ASAS Classification Criteria for Axial Spondyloarthritis (2009) [S15]
	The Classification for Psoriatic Arthritis (CASPAR) Criteria (2006) [S16]
	Diagnostic Criteria for the SAPHO Syndrome (1994) [S17]
ANCA-associated vasculitis	European Medicines Agency Algorithm for ANCA-associated Vasculitis (2007) [S18]
	2022 ACR/EULAR Classification Criteria for ANCA-associated Vasculitis [S19–S21]
Polyarteritis nodosa	The ACR 1990 Criteria for the Classification of Polyarteritis Nodosa [S22]
Giant cell arteritis	2022 ACR/EULAR Classification Criteria for Giant Cell Arteritis [S23]
Polymyalgia rheumatica	Bird Criteria for Polymyalgia Rheumatica (1987) [S24]
Takayasu arteritis	2022 ACR/EULAR Classification Criteria for Takayasu Arteritis [S25]
Relapsing polychondritis	Damiani Diagnostic Criteria for Relapsing Polychondritis (1979) [S26]
IgG4-related disease	The 2019 ACR/EULAR Classification Criteria for IgG4-related Disease [S27]
	The 2020 Revised Comprehensive Diagnostic Criteria for IgG4-related Disease [S28]
Behçet’s disease	The International Criteria for Behçet’s Disease (2014) [S29]
	2016 Japanese Ministry of Health, Labor and Welfare Diagnostic Criteria for Behçet’s Disease [S30]
Still’s disease	Yamaguchi Diagnostic Criteria for Adult-onset Still’s Disease (1992) [S31]

ACR, American College of Rheumatology; ANCA, anti-neutrophil cytoplasmic antibody; ASAS, Assessment of SpondyloArthritis international Society; EULAR, European Alliance of Associations for Rheumatology; SAPHO, synovitis-acne-pustulosis-hyperostosis-osteomyelitis; SLICC, Systemic Lupus International Collaborating Clinics.

Supplementary Table S2. Autoantibody profiles and its coexistence with anti-SMN antibody

Disease	Antibody	Positivity	Coexistence with anti-SMN antibody
Mixed connective tissue disease (n = 30)	Anti-SMN antibody[a]	11 (36.7)	N/A
	Anti-U1-RNP antibody[a]	27 (90.0)	11 (40.7)
	Anti-Sm antibody[a]	5 (16.7)	4 (80.0)
	Anti-ds-DNA antibody[a]	5 (16.7)	3 (60.0)
	Anti-SS-A/B antibody	16 (53.3)	6 (37.5)
Systemic lupus erythematosus (n = 188)	Anti-SMN antibody[a]	20 (10.6)	N/A
	Anti-U1-RNP antibody[a]	40 (21.3)	16 (40.0)
	Anti-Sm antibody[a]	40 (21.3)	19 (47.5)
	Anti-ds-DNA antibody[a]	107 (56.9)	15 (14.0)
	Anti-SS-A/B antibody	118 (62.8)	14 (11.9)
Systemic sclerosis (n = 126)	Anti-SMN antibody[a]	3 (2.4)	N/A
	Anti-centromere antibody	61 (48.4)	0 (0.0)
	Anti-topoisomerase I antibody	35 (27.8)	1 (2.9)
	Anti-RNA polymerase III antibody	9 (7.1)	0 (0.0)
Idiopathic inflammatory myopathy (n = 72)	Anti-SMN antibody[a]	1 (1.4)	N/A
	Anti-aminoacyl-tRNA synthetase antibody	22 (30.6)	0 (0.0)
	Anti-MDA5 antibody	14 (19.4)	0 (0.0)
	Anti-TIF1-γ antibody	8 (11.1)	0 (0.0)
	Anti-Mi2 antibody	3 (4.2)	0 (0.0)
	Other myopathy specific autoantibody[b]	6 (8.3)	0 (0.0)
Primary Sjögren's disease (n = 136)	Anti-SMN antibody[a]	0 (0.0)	N/A
	Anti-SS-A/B antibody	107 (78.7)	0 (0.0)
	Anti-centromere antibody	20 (14.7)	0 (0.0)
Rheumatoid arthritis (n = 91)	Anti-SMN antibody[a]	1 (1.1)	N/A
	Rheumatoid factor	55 (60.4)	0 (0.0)
	Anti-cyclic citrullinated peptide antibody	47 (51.6)	1 (2.1)
ANCA-associated vasculitis (n = 76)	Anti-SMN antibody[a]	0 (0.0)	N/A
Eosinophilic granulomatosis with polyangiitis (n = 25)	Anti-myeloperoxidase/p-ANCA	4 (16.0)	0 (0.0)
	Anti-proteinase 3/c-ANCA	0 (0.0)	N/A
Granulomatosis with polyangiitis (n = 26)	Anti-myeloperoxidase/p-ANCA	17 (65.4)	0 (0.0)
	Anti-proteinase 3/c-ANCA	8 (30.8)	0 (0.0)
Microscopic polyangiitis (n = 25)	Anti-myeloperoxidase/p-ANCA	23 (92.0)	0 (0.0)
	Anti-proteinase 3/c-ANCA	1 (4.0)	0 (0.0)

[a] Antibody positivity was assessed at the time of blood test.

[b] Other autoantibodies include anti-3-hydroxy-3-methylglutaryl-CoA reductase (HMGCR), anti-signal recognition particle (SRP), anti-nuclear matrix protein 2 (NXP2), and anti-RuvBL1/2 antibodies.

ANCA, anti-neutrophil cytoplasmic antibody; MDA5, melanoma differentiation-associated protein 5; RNA, ribonucleic acid; RNP, ribonucleoprotein; SMN, survival of motor neuron; TIF1, transcriptional intermediary factor 1.

Supplementary Table S3. Cumulative clinical characteristics of MCTD patients stratified by anti-SMN antibodies

	**Negative, n = 19 (SMN ≤ 1,050 MFI)**	**Positive, n = 11 (SMN > 1,050 MFI)**	***P*-value**
**SLE manifestations, n (%)**	19 (100.0)	11 (100.0)	N/A
Polyarthritis, n (%)	13 (68.4)	10 (90.9)	.339
Lymphadenopathy, n (%)	9 (47.3)	8 (72.7)	.259
Malar rash, n (%)	6 (31.6)	3 (27.3)	> .999
Pericarditis or pleuritis, n (%)	6 (31.6)	2 (18.2)	.672
Leukopenia (≤ 4,000 /μL) or thrombocytopenia (≤ 100,000 /μL), n (%)	11 (57.9)	5 (45.5)	.707
**SSc manifestations, n (%)**	14 (73.7)	11 (100.0)	.129
Sclerodactyly, n (%)	11 (57.9)	7 (63.6)	> .999
Interstitial lung disease, n (%)	7 (36.8)	10 (90.9)	.013
Esophageal dysmotility or dilatation, n (%)	5 (26.3)	2 (18.2)	.952
**PM/DM manifestations, n (%)**	7 (36.8)	4 (36.4)	> .999
Myositis, n (%)	7 (36.8)	4 (36.4)	> .999
Elevated levels of myogenic enzymes, n (%)	7 (36.8)	4 (36.4)	> .999
All manifestations of SLE, SSc and PM/DM, n (%)	5 (26.3)	4 (36.4)	.687

N/A, not applicable; MCTD, mixed connective tissue disease; MFI, median fluorescence intensity; PM/DM, polymyositis/dermatomyositis; SLE, systemic lupus erythematosus; SMN, survival of motor neuron; SSc, systemic sclerosis.

Supplementary Table S4. Clinical characteristics of newly onset SLE patients stratified by anti-SMN antibodies

	**Negative, n = 49 (SMN ≤ 1,050 MFI)**	**Positive, n = 12 (SMN > 1,050 MFI)**	***P*-value**
Age at blood test (years), mean (SD)	49.5 (18.5)	36.9 (17.9)	.046
Disease duration (months), median (IQR)	0.0 (0.0–2.0)	0.5 (0.0–2.2)	.721
Female, n (%)	40 (81.6)	10 (83.3)	> .999
Body mass index (kg/m^2), median (IQR)	20.0 (18.0–22.0)	19.5 (18.0–22.2)	.985
White blood cell counts (/µL), median (IQR)	4,300.0 (2,700.0–5,200.0)	2,700.0 (2,200.0–3,525.0)	.035
Lymphocyte (/µL), median (IQR)	893.0 (535.5–1,070.0)	754.0 (479.9–829.5)	.162
Hemoglobin (g/dL), median (IQR)	10.7 (9.5–11.9)	9.4 (8.3–9.9)	.038
Platelet (10^3/µL), median (IQR)	219.0 (123.0–281.0)	133.0 (8.2–187.0)	.071
eGFR (mL/min/1.73 m^2), median (IQR)	84.0 (70.0–110.0)	75.5 (58.0–92.0)	.249
C-reactive protein (mg/L), median (IQR)	5.9 (0.7–13.6)	1.4 (0.3–13.5)	.268
Immunoglobulin G (mg/dL), median (IQR)	1,959.0 (1,500.8–2,505.2)	2,334.0 (1,830.8–2,826.5)	.216
Complement 3 (mg/dL), median (IQR)	65.0 (42.8–90.8)	29.0 (28.0–35.2)	.001
Complement 4 (mg/dL), median (IQR)	11.5 (5.0–16.2)	4.0 (3.0–8.5)	.005
Immune complex-C1q (µg/mL), median (IQR)	1.9 (0.0–6.1)	7.8 (4.5–12.4)	.043
Anti-SMN antibody titer (MFI), median (IQR)	160.0 (128.0–264.0)	1,931.0 (1,645.8–4,051.5)	< .001
Positivity for anti-recombinant U1-RNP antibody, n (%)	6 (12.2)	11 (91.7)	< .001
Positivity for anti-recombinant Sm antibody, n (%)	8 (16.3)	12 (100.0)	< .001
Positivity for anti-ds-DNA antibody, n (%)	39 (79.6)	10 (83.3)	> .999
Anti-ds-DNA antibody titer (IU/mL), median (IQR)	20.6 (7.4–46.6)	260.0 (97.2–400.0)	.001
Positivity for anti-SS-A antibody, n (%)	29 (59.2)	8 (66.7)	.749
Positivity for anti-cardiolipin antibody, n (%)	17 (34.7)	8 (66.7)	.056
**Active manifestations**			
Fever, n (%)	16 (32.7)	7 (58.3)	.182
Mucocutaneous, n (%)	23 (46.9)	7 (58.3)	.534
Neuropsychiatric, n (%)	10 (20.4)	1 (8.3)	.438
Musculoskeletal, n (%)	23 (46.9)	7 (58.3)	.534
Cardiorespiratory, n (%)	6 (12.2)	1 (8.3)	> .999
Serositis, n (%)	14 (28.6)	7 (58.3)	.088
Gastrointestinal, n (%)	3 (6.1)	3 (25.0)	.084
Nephritis, n (%)	15 (30.6)	9 (75.0)	.008
Classification of renal pathology (III/IV, III/IV + V, V), n (%)	5 (10.2), 1 (2.0), 2 (4.1)	6 (50.0), 1 (8.3), 1 (8.3)	.004
Hemolytic anemia, n (%)	4 (8.2)	4 (33.3)	.041
SLEDAI, median (IQR)	14.0 (9.0–20.0)	26.5 (19.8–31.9)	.008
SLICC/ACR damage index at baseline, median (IQR)	0.0 (0.0–0.0)	0.0 (0.0–0.0)	.883
Concurrent rheumatic and musculoskeletal diseases, n (%)	19 (38.8)	4 (33.3)	> .999
Concurrent Sjögren's disease, n (%)	14 (28.6)	4 (33.3)	.736
Concurrent antiphospholipid syndrome, n (%)	4 (8.2)	0 (0.0)	.576

eGFR, estimated glomerular filtration rate; IQR, interquartile range; MFI, median fluorescence intensity; RNP, ribonucleoprotein; SD, standard deviation; SLE, systemic lupus erythematosus; SLEDAI, SLE Disease Activity Index; SLICC/ACR, Systemic Lupus International Collaborating Clinics/American College of Rheumatology; SMN, survival of motor neuron.

Reference

S1. Tanaka Y, Kuwana M, Fujii T, Kameda H, Muro Y, Fujio K, et al. 2019 Diagnostic criteria for mixed connective tissue disease (MCTD): From the Japan research committee of the ministry of health, labor, and welfare for systemic autoimmune diseases. Mod. Rheumatol. 31 (2021) 29–33. https://doi.org/10.1080/14397595.2019.1709944.

S2. Petri M, Orbai AM, Alarcõn GS, Gordon C, Merrill JT, Fortin PR, et al. Derivation and validation of the Systemic Lupus International Collaborating Clinics classification criteria for systemic lupus erythematosus. Arthritis. Rheum. 64 (2012) 2677–86. https://doi.org/10.1002/art.34473.

S3. Aringer M, Costenbader K, Daikh D, Brinks R, Mosca M, Ramsey-Goldman R, et al. 2019 European League Against Rheumatism/American College of Rheumatology Classification Criteria for Systemic Lupus Erythematosus. Arthritis. Rheum. 71 (2019) 1400–1412. https://doi.org/10.1002/art.40930.

S4. Asano Y, Jinnin M, Kawaguchi Y, Kuwana M, Goto D, Sato S, et al. Diagnostic criteria, severity classification and guidelines of systemic sclerosis. J. Dermatol. 45 (2018) 633–91. https://doi.org/10.1111/1346-8138.14162.

S5. Van Den Hoogen F, Khanna D, Fransen J, Johnson SR, Baron M, Tyndall A, et al. 2013 classification criteria for systemic sclerosis: an American College of Rheumatology/European League Against Rheumatism collaborative initiative. Arthritis. Rheum. 65 (2013) 2737–47. https://doi.org/10.1002/art.38098.

S6. Bohan A, Peter JB. Polymyositis and dermatomyositis (first of two parts). N. Engl. J. Med. 292 (1975) 344–7.

S7. Euwer RL, Sontheimer RD. Amyopathic dermatomyositis (dermatomyositis sine myositis). Presentation of six new cases and review of the literature. J. Am. Acad. Dermatol. 24 (1991) 959–66.

S8. Ministry of Health, Labor and Welfare, Japan. Diagnostic criteria for polymyositis and dermatomyositis (PM/DM). https://www.mhlw.go.jp/stf/seisakunitsuite/bunya/0000062437.html Accessed 16 April 2025.

S9. Lundberg IE, Tjärnlund A, Bottai M, Werth VP, Pilkington C, Visser M de, et al. 2017 European League Against Rheumatism/American College of Rheumatology classification criteria for adult and juvenile idiopathic inflammatory myopathies and their major subgroups. Ann. Rheum. Dis. 76 (2017) 1955–64. https://doi.org/10.1136/annrheumdis-2017-211468.

S10. Fujibayashi T, Sugai S, Miyasaka N, Hayashi Y, Tsubota K. Revised Japanese criteria for Sjögren's syndrome (1999): Availability and validity. Mod. Rheumatol. 14 (2004) 425–34. https://doi.org/10.1007/s10165-004-0338-x.

S11. Shiboski CH, Shiboski SC, Seror R, Criswell LA, Labetoulle M, Lietman TM, et al. 2016 American College of Rheumatology/European League Against Rheumatism Classification Criteria for Primary Sjögren's Syndrome: A Consensus and Data-Driven Methodology Involving Three International Patient Cohorts. Arthritis. Rheum. 69 (2017) 35–45. https://doi.org/10.1002/art.39859.

S12. Arnett FC, Edworthy SM, Bloch DA, Mcshane DJ, Fries JF, Cooper NS, et al. The american rheumatism association 1987 revised criteria for the classification of rheumatoid arthritis. Arthritis. Rheum. 31 (1988) 315–24. https://doi.org/10.1002/art.1780310302.

S13. Aletaha D, Neogi T, Silman AJ, Funovits J, Felson DT, Bingham CO, et al. 2010 Rheumatoid arthritis classification criteria: An American College of Rheumatology/European League Against Rheumatism collaborative initiative. Arthritis. Rheum. 62 (2010) 2569–81. https://doi.org/10.1002/art.27584.

S14. Linden S Van Der, Valkenburg HA, Cats A. Evaluation of Diagnostic Criteria for Ankylosing Spondylitis. Arthritis. Rheum. 27 (1984) 361–8. https://doi.org/10.1002/art.1780270401.

S15. Rudwaleit M, Van Der Heijde D, Landewé R, Listing J, Akkoc N, Brandt J, et al. The development of Assessment of SpondyloArthritis international Society classification criteria for axial spondyloarthritis (part II): Validation and final selection. Ann. Rheum. Dis. 68 (2009) 777–83. https://doi.org/10.1136/ard.2009.108233.

S16. Taylor W, Gladman D, Helliwell P, Marchesoni A, Mease P, Mielants H. Classification criteria for psoriatic arthritis: Development of new criteria from a large international study. Arthritis. Rheum. 54 (2006) 2665–73. https://doi.org/10.1002/art.21972.

S17. Kahn MF, Khan MA. The SAPHO syndrome. Bailliere's Clinical Rheumatology 8 (1994) 333–62.

S18. Watts R, Lane S, Hanslik T, Hauser T, Hellmich B, Koldingsnes W, et al. Development and validation of a consensus methodology for the classification of the ANCA-associated vasculitides and polyarteritis nodosa for epidemiological studies. Ann. Rheum. Dis. 66 (2007) 222–7. https://doi.org/10.1136/ard.2006.054593.

S19. Grayson PC, Ponte C, Suppiah R, Robson JC, Craven A, Judge A, et al. 2022 American college of rheumatology/European alliance of associations for rheumatology classification

criteria for eosinophilic granulomatosis with polyangiitis. Ann. Rheum. Dis. 81 (2022) 309–14. https://doi.org/10.1136/annrheumdis-2021-221794.

S20. Robson JC, Grayson PC, Ponte C, Suppiah R, Craven A, Judge A, et al. 2022 American college of rheumatology/European alliance of associations for rheumatology classification criteria for granulomatosis with polyangiitis. Ann. Rheum. Dis. 81 (2022) 315–20. https://doi.org/10.1136/annrheumdis-2021-221795.

S21. Suppiah R, Robson JC, Grayson PC, Ponte C, Craven A, Khalid S, et al. 2022 American college of rheumatology/European alliance of associations for rheumatology classification criteria for microscopic polyangiitis. Ann. Rheum. Dis. 81 (2022) 321–6. https://doi.org/10.1136/annrheumdis-2021-221796.

S22. Lightfoot RW, Michel BA, Bloch DA, Hunder GG, Zvaifler NJ, McShane DJ, et al. The American college of rheumatology 1990 criteria for the classification of polyarteritis nodosa. Arthritis. Rheum. 33 (1990) 1088–93. https://doi.org/10.1002/art.1780330805.

S23. Ponte C, Grayson PC, Robson JC, Suppiah R, Gribbons KB, Judge A, et al. 2022 American College of Rheumatology/EULAR Classification Criteria for Giant Cell Arteritis. Arthritis. Rheum. 74 (2022) 1881–9. https://doi.org/10.1002/art.42325.

S24. Bird HA, Esselinckx W, Dixon SAJ, Mowat AG, Wood PH. An evaluation of criteria for polymyalgia rheumatica. Ann. Rheum. Dis. 38 (1979) 434–9. https://doi.org/10.1136/ard.38.5.434.

S25. Grayson PC, Ponte C, Suppiah R, Robson JC, Gribbons KB, Judge A, et al. 2022 American College of Rheumatology/EULAR classification criteria for Takayasu arteritis. Ann. Rheum. Dis. 81 (2022) 1654–60. https://doi.org/10.1136/ard-2022-223482.

S26. Damiani JM, Levine HL. Relapsing polychondritis — report of ten cases. Laryngoscope 89 (1979) 929–46. https://doi.org/10.1288/00005537-197906000-00009.

S27. Wallace ZS, Naden RP, Chari S, Choi H, Della-Torre E, Dicaire JF, et al. The 2019 American College of Rheumatology/European League Against Rheumatism Classification Criteria for IgG4-Related Disease. Arthritis. Rheum. 72 (2020) 7–19. https://doi.org/10.1002/art.41120.

S28. Umehara H, Okazaki K, Kawa S, Takahashi H, Goto H, Matsui S, et al. The 2020 revised comprehensive diagnostic (RCD) criteria for IgG4-RD. Mod. Rheumatol. 31 (2021) 529–33. https://doi.org/10.1080/14397595.2020.1859710.

S29. Davatchi F, Assaad-Khalil S, Calamia KT, Crook JE, Sadeghi-Abdollahi B, Schirmer M, et al. The International Criteria for Behçet's Disease (ICBD): A collaborative study of 27 countries on the sensitivity and specificity of the new criteria. J. Eur. Acad. Dermatol. Venereol. 28 (2014)338–47. https://doi.org/10.1111/jdv.12107.

S30. Ministry of Health, Labor and Welfare, Japan. Diagnostic criteria for Behçet's Disease. https://www.mhlw.go.jp/stf/seisakunitsuite/bunya/0000062437.html Accessed 16 April 2025.

S31. Yamaguchi M, Ohta A, Tsunematsu T, Kasukawa R, Mizushima Y, Kashiwagi H, et al. Preliminary Criteria for Classification of Adult Still's Disease. J. Rheumatol. 19 (1992) 424–54.
