# Supplementary material for: Anti-survival of motor neuron antibodies in rheumatic and musculoskeletal diseases: prevalence, clinical associations, and biomarker potential, with novel insights into disease activity in SLE
Source: Inflamm Regen. 2025 Dec 2;46:1. doi: 10.1186/s41232-025-00399-w (PMC12777490; doi:10.1186/s41232-025-00399-w)
Supplement: Supplementary file 2 — Supplementary Material 2. [file 41232_2025_399_MOESM2_ESM.pdf]

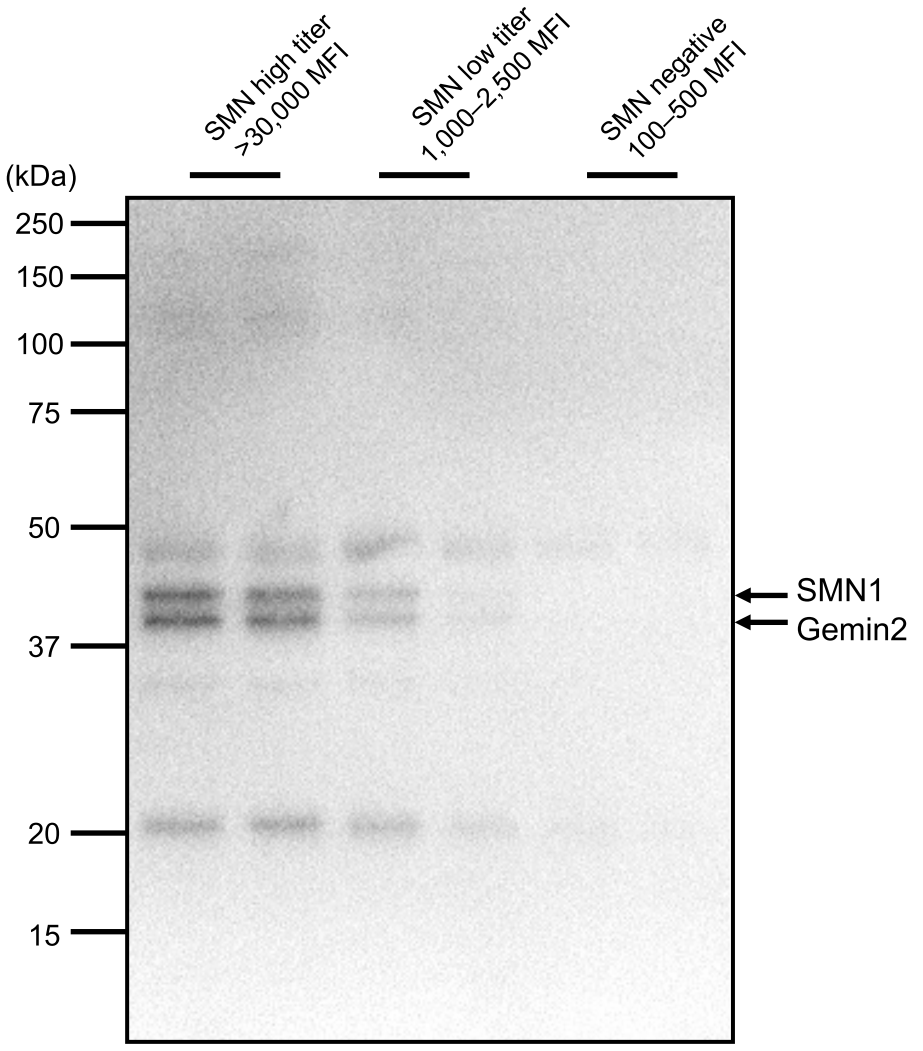

Supplementary Figure S1. Immunoprecipitation and Western blotting of SMN antigens.
Protein G magnetic beads bound to immunoglobulin G from 6 individuals were incubated with SBP- and His-tagged SMN complexes, resolved by SDS-PAGE, and blotted with Strep-Tactin HRP conjugate. The arrows indicate the expected molecular weight of SMN and Gemin2.

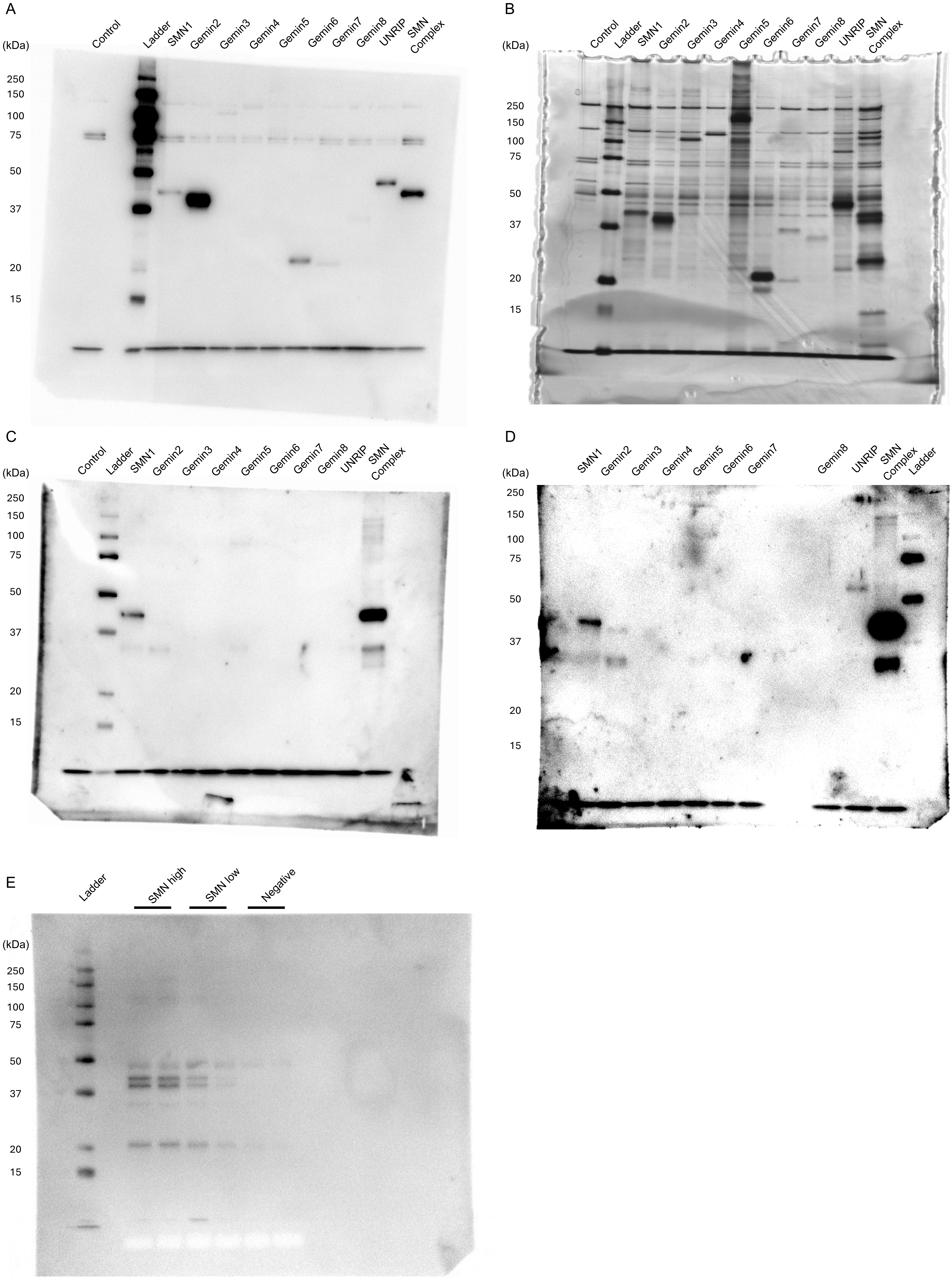

Supplementary Figure S2. Full-length original gel and blot images.
Control indicates cell lysates without vector transfection. Corresponds to (A) Figure 1A, (B) Figure 1B, (C) Figure 2A, (D) Figure 2B, and (E) Suppromentary Figure S1.
